# Pregnancy-Associated Breast Cancer in Taiwanese Women: Potential Treatment Delay and Impact on Survival

**DOI:** 10.1371/journal.pone.0111934

**Published:** 2014-11-21

**Authors:** Ya-Ling Yang, K. Arnold Chan, Fon-Jou Hsieh, Li-Yun Chang, Ming-Yang Wang

**Affiliations:** 1 Department of Nursing, College of Medicine, National Taiwan University, No. 1, Jen-Ai Road, Sec 1, Taipei 10051, Taiwan; 2 Department of Medical Research, National Taiwan University Hospital, No. 7, Chung-Shan South Road, Taipei 10051, Taiwan; 3 Graduate Institute of Oncology, College of Medicine, National Taiwan University, No. 1, Jen-Ai Road, Sec 1, Taipei 10051, Taiwan; 4 Department of Obstetrics and Gynecology, College of Medicine, National Taiwan University, No. 1, Jen-Ai Road, Sec 1, Taipei 10051, Taiwan; 5 Department of Obstetrics and Gynecology, National Taiwan University Hospital, No. 7, Chung-Shan South Road, Taipei 10051, Taiwan; 6 Department of Surgery, National Taiwan University Hospital, No. 7, Chung-Shan South Road, Taipei 10051, Taiwan; Taipei Medical University, Taiwan

## Abstract

This study investigated the clinicopathologic characteristics and survival of women diagnosed with pregnancy-associated breast cancer (PABC) in Taiwan. PABC is defined as breast cancer diagnosed during pregnancy or within 1 year after obstetric delivery. Our sample of PABC patients (*N* = 26) included all patients diagnosed at a major medical center in northern Taiwan from 1984 through 2009. Among these patients, 15 were diagnosed during pregnancy and 11 were diagnosed within 1 year after delivery. The comparison group included 104 patients within the same age range as the PABC patients and diagnosed with breast cancer not associated with pregnancy from 2004 through 2009 at the same hospital. Patients' initiating treatment delayed, 5-year and 10-year overall survival were delineated by stratified Kaplan-Meier estimates. Patients' characteristics were associated with initiating treatment delayed was evaluated with multivariate proportional hazards modeling. Antepartum PABC patients were younger and had longer time between diagnosis and treatment initiation than postpartum PABC patients. The predictor of treatment delayed was including birth parity, cancer stage, and pregnancy. The PABC group had larger tumors, more advanced cancer stage, and tumors with less progesterone receptor than the comparison group. The antepartum PABC patients had higher mortality than postpartum PABC and comparison groups within 5 years after diagnosis. Based on these results, we confirmed that pregnant women with breast cancer were more likely to delay treatment. Therefore, we recommend that breast cancer screening should be integrated into the prenatal and postnatal routine visits for early detection of the women's breast problems.

## Introduction

Breast cancer is the most common malignancy among women in Taiwan and its incidence has been rising in Taiwan over the past two decades [1]–[3]. Furthermore, breast cancer patients in Taiwan tend to be much younger than their counterparts in western countries [2], [4]. Early-onset breast cancer, defined as diagnosis before age 45, comprised 26.4% of all breast cancer patients in Taiwan in 2007 [3], and this sizable patient population represents women of childbearing age. Although the incidence of pregnancy-associated breast cancer (PABC) is relatively low in Asia [5], [6], more women are postponing their first term pregnancy age after 30 s, and incidence of PABC is expected to increase [7]. Unfortunately, there is a knowledge gap for understanding whether pregnancy and other prognostic factors affect the survival of women with PABC.

Pregnancy-associated breast cancer (PABC), defined as breast cancer diagnosed during gestation or within 1 year after delivery [8], [9], may have a devastating effect not only on the mother but also on the fetus. Although PABC is rare, with reported frequencies of 1 in 1,000 to 3 in 10,000 pregnancies [8]–[12], its diagnosis may be delayed by difficulty interpreting clinical examinations and mammography due to increased breast density in pregnant and breastfeeding women [13], [14]. Treatment of PABC poses specific challenges as the regimen may harm the fetus [15], and while delayed or under-treatment may be less harmful to the fetus, treatment efficacy for the mother may be suboptimal [5]. Another complication of treatment for PABC is that the growth of breast cancer cells may be enhanced by hormonal and immunologic changes in pregnancy; thus, a delay of just 1 to 2 months in initiating therapy can increase the likelihood of lymph node metastasis [13]–[15]. However, this potential deleterious effect has not been well studied.

Due to delay in diagnosis, PABC patients tend to present at a more advanced stage at initial diagnosis and have a higher risk of metastatic disease than non-pregnant women, resulting in worse prognosis [5], [6], [16]–[18]. In contrast, some previous studies reported that pregnancy was not found to be an independent factor for biologically more aggressive cancer and poorer survival among breast cancer patients, after controlling for cancer stage and age [6], [9], [19]–[21]. Asian women had a lower risk of PABC mortality compared with other ethnicity, but a higher proportion of PABC was reported to be of Hispanic ethnicity and Asian/Pacific Islander (37.9%) compared with age-matched non-pregnant control cases (30.9%) and a lower proportion to be non-Hispanic white by a US study finding which was linked with the California Cancer Registry (1991–1999) and California Patient Discharge Data set [16]. Younger Taiwanese women (30–34 years old) had similar incidence of breast cancer as Caucasians American women (24.91 per 100,000 Taiwanese compared to 24.2 per 100,000 in the USA based on 2006) [2], but little is known about PABC in Taiwan. We conducted a retrospective study of PABC patients diagnosed at a single medical center in Taiwan to evaluate the demographic and clinical factors associated with risk for more advanced disease at diagnosis, delayed treatment, and increased mortality.

## Materials and Methods

### Sample and setting

For this retrospective study, the PABC group consisted of all women diagnosed with PABC from 1984 through to 2009 at a 2000-bed, urban, tertiary medical center in northern Taiwan. We collected the data only from one site, for two reasons. Firstly, this medical center has a well-coordinated OBGY department and breast center to provide continuous perinatal care and breast oncologic treatment for PABC patients. Thus, we could collect comprehensive maternal-fetal health, breast surgery and oncological treatment records. Secondly, we had compared the difference in the breast cancer incidence of young ages (age 20∼29, 30∼39, and 40∼49) between this medical center and the Taiwan Cancer Data Base (TCDB) from 2005 to 2009, with differences between the two of less than 3% in each group, suggesting our sample is fairly representative.

PABC was defined as a diagnosis of invasive breast cancer during pregnancy or within 1 year after delivery. Women diagnosed with breast cancer during the 9 months preceding delivery were defined as antepartum cases, whereas women diagnosed with PABC within 12 months after delivery were defined as postpartum cases. The comparison group (four times the number of patients in the PABC group) was randomly selected from women diagnosed at the same hospital with their first non-pregnancy-associated breast cancer, within a similar age range (25–44 years) as the PABC group, and underwent cancer surgery from 2004 through 2009.

### Data collection

Data were collected from medical records of patients in the PABC and comparison groups. Extracted data elements included age at diagnosis, cancer stage at diagnosis, tumor size (longest diameter), lymph node involvement, treatment (surgery, chemotherapy and/or radiation), hormone receptor status, and survival. Cancer staging was evaluated according to American Joint Committee on Cancer staging (AJCC) 6th edition [22]. Tumor expression of estrogen receptor (ER), progesterone receptor (PR), and human epidermal growth factor receptor 2 (HER-2/neu) was detected by immunohistochemistry [22]. For ER and PR, a nuclear stain was considered positive. HER-2/neu protein staining was graded from 0 to 3+ using the HercepTest method (Dako Corporation, Carpinteria, CA). Grade 3 + was regarded as HER-2 positive or over-expression, whereas grades 0, 1+ and 2+ were regarded as HER-2/negative. The study was approved by the Research Ethics Committee of National Taiwan University Hospital (200706039R). All of the participants were legal adults, and any identifying information was removed prior to data analysis, ensuring participants' privacy.

### Data analysis

Data were analyzed using R version 2.15.2 (R Foundation for Statistical Computing, Vienna, Austria), with two-tailed p value≤0.05 considered statistically significant. In univariate comparisons, potential prognostic factors were examined among the PABC antepartum and postpartum subgroups and the non-PABC group using the Mann-Whitney Wilcoxon test for categorical variables (e.g., cancer stage, hormone receptor status) and the Pearson Chi-square test for continuous variables (e.g., age, tumor size). Survival was analyzed by comparing the time to treatment and overall survival time with Kaplan-Meier curves and log-rank test. Time to treatment was defined as the time from confirmed diagnosis to initiation of any treatment regimen. Overall survival was defined as the time from diagnosis to death of any cause. Estimated effects of prognostic factors on time-to-treatment were compared between the two PABC subgroups and between the PABC and non-PABC group using the multivariate Cox proportional hazards model [23]. Regression models were used to estimate the strength of association for individual factors as expressed in hazard ratios (HRs) and their corresponding 95% confidence intervals (CIs).

## Results

Of the 26 PABC patients diagnosed at a medical center from 1984 through 2009, 15 were antepartum cases and 11 were postpartum cases. All of their pathological diagnosis was infiltrating duct carcinoma-not otherwise specified (NOS), except one antepartum case of intraductal papillary-mucinous carcinoma-invasive, and one postpartum case of carcinoma-NOS. A comparison group of 104 women (4×26) was randomly selected from 2,904 women between 25 and 44 years old when diagnosed with non-pregnancy-related invasive breast cancer (88.5% were infiltrating duct carcinoma-NOS, and others as follow mucinous adenocarcinoma, carcinoma-NOS, comedocarcinoma-NOS, intraductal papillary adenocarcinoma with invasion, infiltrating duct and lobular carcinoma, lobular carcinoma-NOS, paget disease and intraductal carcinoma of breast, metaplastic carcinoma-NOS, and phyllodes tumor-malignant) and operated for breast cancer at the same hospital from 2004 through 2009.

### Characteristics of PABC antepartum and postpartum patients

Patients in the PABC antepartum subgroup were younger (median age = 33 years) at breast cancer diagnosis than those in the postpartum subgroup (median age = 37 years) (Table 1). Within the antepartum subgroup, ten patients were nulliparous women (median age = 34 years) and five were multipara (median age of their first pregnancy = 28 years). The median fetal gestational age at diagnosis was 29 weeks (mean = 25, range = 6–37) and the median delay in initiating treatment (until after delivery) was 33 weeks (range = 30–40). To initiate early breast cancer treatment, four patients chose abortion (median = 20 weeks, range = 14–20). In the postpartum subgroup, the median time after delivery for breast cancer diagnosis was 6 months (range = 2–11 months). Four patients in this group had found breast abnormalities during pregnancy but thought they were normal pregnancy-associated changes. Within the postpartum subgroup, eight patients were nulliparous mothers (median age = 32 years) and three multiparous patients whose first pregnant median age were same as the antepartum subgroup's. These two PABC subgroups did not differ significantly with regard to time between self-reported onset of symptoms/signs to confirmed diagnosis, age at menarche, age at first pregnancy, gravida, and number of childbirths. However, the median tumor size of patients in the antepartum PABC was larger than that of postpartum patients, but not statistically different. Other clinical and pathological characteristics of these subgroups were similar (Table 1).

**Table 1:** Demographic and clinical characteristics of patients with and without pregnancy-associated breast cancer (PABC).

Characteristic	PABC subjects				Non-PABC subjects	
	Total (N = 26)	Antepartum (n = 15)	Postpartum (n = 11)	Comparison^a^	(N = 104)	Comparison^b^
Age at diagnosis, years (median, range)	34.0 (25–41)	33.0 (25.0–40)	37.0 (30–41)	<0.01	40 (25–44)	<0.001
Gestational age at delivery (median, range)	36.0 (8–41)	32.0 (8–41)	38.0 (16–41)	<0.05		
Tumor size, cm (median, range)	3.0 (1.3–10)	3.5 (1.5–10)	3.0 (1.3–5)	*NS*	2 (0.1–8.8)	<0.001
Cancer stage (n, %)				*NS*		<0.05
0	0	0	0		18 (17.3)	
I	5 (19.2)	2 (13.3)	3 (27.3)		24 (23.1)	
II	15 (57.7)	8 (53.3)	7 (63.6)		38 (36.5)	
III	4 (15.4)	3 (20.0)	1 (09.1)		19 (18.3)	
IV	2 (7.7)	2 (13.3)	0		5 (4.8)	
Surgical method (n, %)				*NS*		*NS*
None	1 (3.9)	1 (6.7)	0		5 (4.8)	
Modified radical mastectomy	14 (53.8)	10 (66.7)	4 (36.4)		43 (41.4)	
Lumpectomy/partial mastectomy	11 (42.3)	4 (26.7)	7 (63.6)		56 (53.8)	
Pre-op neoadjuvant (n, %)	5 (19.2)	5 (33.3)	0		7 (6.7)	
Lymph node involvement (n, %)	11 (42.3)	6 (40.0)	5 (45.5)		50 (48.1)	*NS*
Hormone receptor (n, %)						
ER positive	17/9 (65.4)	9 (60.0)	8 (72.7)		51/16 (76.1)	*NS*
PR positive	9/17 (34.6)	5 (33.3)	4 (36.4)		40/26 (60.6)	<0.01
Her-2/new positive	10/11 (47.6)	6 (40.0)	4 (36.4)		13/53 (19.7)	*NS*
Self-reported onset of symptoms/signs before diagnosis, weeks (median)		4	1	*NS*		
Menarche, years (median)		13	13	*NS*		
Age at first pregnancy, years (median)		29	33	*NS*		
Gravida (median)		2	2	*NS*		
Nulliparous (n)		10	8			
Deliveries (median)		1	1	*NS*		

NS =  nonsignificant.

Comparison between antepartum and postpartum PABC subjects.

Comparison between PABC and non-PABC subjects.

The surgical approach for the majority of antepartum PABC patients (10/15) was modified radical mastectomy, whereas seven of 11 postpartum PABC patients had breast conserving surgery. In the antepartum PABC subgroup, six patients were initially treated surgically, with mastectomy for three patients delayed a median of 4 weeks after diagnosis to receive caesarean section at the same time (median gestation  = 37 weeks). Two antepartum PABC patients underwent surgery at 24 weeks gestation and one at 31weeks gestation. Fetal compromise was not associated with preterm labor.

Five antepartum PABC patients were initially treated with neoadjuvant chemotherapy before surgery, and all postpartum patients were initially treated surgically. For all five patients receiving neoadjuvant chemotherapy, their diagnosis was confirmed in the third trimester (median and mean gestation = 32 weeks); for three of these patients, their breast cancers were classified at stages I, II, and III, with two patients at stage IV. Administration of drugs for neoadjuvant chemotherapy was delayed 1 week to 2 months until fetuses had matured (median gestational age = 36 weeks; mean = 35.6 weeks). All five infants exposed in utero to chemotherapy agents were apparently healthy, with no major defect found at birth.

### Comparison of antepartum and postpartum PABC patients' treatment delay and survival

Antepartum PABC patients had a significantly longer median time between diagnosis and initial treatment (surgery/chemotherapy) than that for the postpartum subgroup (median = 21 days; range = 0–89 vs. median = 6; range = 2–15, respectively; log-rank test, *p* = 0.01). The association between patient characteristics and time between PABC diagnosis and initial treatment was evaluated by the stepwise method of multivariate analysis in the Cox proportional hazards model. We found that the risk for delayed treatment initiation was lower for patients diagnosed with postpartum PABC (antepartum patients' HR = 1) (HR = 0.18; 95% CI = 0.06–0.56) and who were multipara (nullipara HR = 1) (HR = 0.31; 95% CI = 0.11–0.86). Furthermore, 23.1% (6/26) of patients diagnosed with early stage (Stage I) PABC had 3.85 times greater risk of delayed initial treatment than patients diagnosed at other stages (Table 2).

**Table 2:** Multivariate Analyses of Predictors of Treatment Delay using the Cox Model.

Covariate	Hazard Ratio	95% Confidence Interval	*p*
1. Predictors of treatment delay (diagnosis to surgery or chemotherapy) in the PABC group			
Postpartum vs antepartum	0.18	0.06–0.56	<0.001
Stage I vs other stages	3.84	1.22–12.50	<0.05
Multipara vs nullipara	0.31	0.11–0.86	<0.05
Adjusted generalized *R* ^2^ = 0.50.			
2. Predictors of treatment delay among two PABC subgroups and non-PABC group			
Postpartum PABC vs antepartum PABC and non-PABC	0.51	0.26–0.98	<0.05
Antepartum PABC vs postpartum PABC and non-PABC	2.04	1.12–3.70	<0.05
Adjusted generalized *R* ^2^ = 0.15			

1. Independent variables included PABC subgroup (postpartum/antepartum), age at diagnosis, tumor size, cancer stage (I–IV), lymph node involvement, expression of ER, PR, and Her-2/neu (yes/no), and surgery (none, modified radical mastectomy, partial mastectomy) with stepwise method of multivariate analysis in the Cox proportional hazard model.

2. Independent variables included antepartum PABC, postpartum PABC, non-PABC, age at diagnosis, tumor size, cancer stage (I–IV), lymph node involvement, expression of ER, PR, and Her-2/neu (yes/no), and surgery (none, modified radical mastectomy, partial mastectomy) with stepwise method of multivariate analysis in the Cox proportional hazard model.

**Figure 1:**
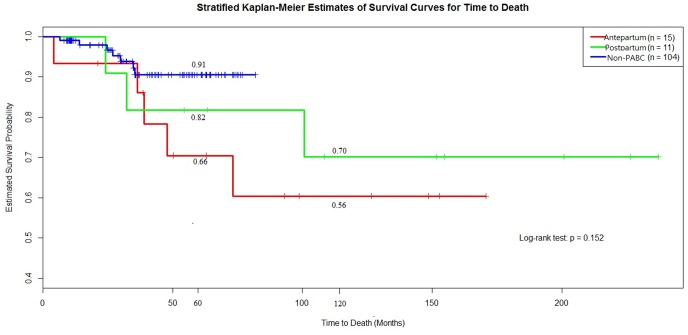
The 5-year survival curve among antepartum PABC, postpartum PABC and non-PABC; 10-year survival curve between antepartum PABC and postpartum PABC. 1. The 5-year survival estimate among three groups, Log-rank test, 0.15. 2. The 10-year survival estimate between PABC two subgroups Log-rank test, 0.45.

Review of the medical record showed that eight PABC patients (30.8%; five antepartum and three postpartum patients) died of breast cancer; of these eight patients, half (three antepartum patients and one postpartum patient) had had recurrences within 1.5 years after diagnosis. Of the eight dead patients, six were nullipara (a half of each subgroup) who had their first pregnant mean age at 32 years (median = 29 years) and two multiparous patients were in the antepartum subgroup (age of first pregnant, mean = 28 years). Of the remaining 18 PABC patients, two had censored data, but both had survived at least 5 years. Fourteen patients (53.8%) survived with relapse-free status at the end of 2009. Two patients had second cancer diagnosis during follow up periods (one patient had thyroid cancer after 8 years and the other patient had lung cancer after 11 years follow-up). Cumulative 5- and 10-year survival rates estimated by the Kaplan-Meier method were 65.7% and 56.4%, respectively in the antepartum subgroup and 81.8% and 70.1%, respectively in the postpartum subgroup (Figure 1). The two subgroups did not differ in overall survival estimated by the stratified Kaplan-Meier method (log-rank test, *p* = 0.45).

### Characteristics and comparison of patients with PABC and non-PABC

Comparison of characteristics of patients diagnosed with PBAC and non-pregnancy-related breast cancer (non-PABC) showed differences in their tumor pathological characteristics. PABC patients had larger primary tumors (median = 3.0 cm; range = 1.3–10) than non-PABC patients (Table 1). But there was no difference in the tumor pathology between these two groups. Among the 26 PABC patients, none had a cancer at the 0 stage, but 18 (17.3%) of 104 patients in the comparison group had 0 stage cancers. The PABC and non-PABC patients did not differ in their surgical method of treatment, or in the proportion with lymph node involvement at time of surgery (42% vs. 48%). Fewer PABC patients had tumors expressing ER and PR than the comparison group, but this difference was only significant for PR expression. A higher proportion of PABC patients than non-PABC patients had tumors over-expressing HER-2/neu (Table 1).

The time between diagnosis and treatment initiation did not differ significantly between PABC patients (mean = 25.9 days; median = 10 days) and non-PABC patients (mean = 14.7 days; median = 12 days) with the Kaplan-Meier estimate (log-rank test, *p* = 0.07). We also evaluated the association between patient characteristics and the time between diagnosis and initial treatment among the PABC antepartum, PABC postpartum, and non-PABC patients using the stepwise method of analysis in the Cox proportional hazards model. The results showed that postpartum PABC patients (vs. antepartum PABC and non-PABC patients) and antepartum PABC patients had 0.51 and 2.04 times the risk of a delay in their initial treatments, respectively (Table 2).

The cumulative 5-year survival rates estimated with the Kaplan-Meier method for the antepartum PABC, postpartum PABC, and non-PABC comparison groups were 65.7%, 81.8%, and 90.5%, respectively. However, overall survival did not differ significantly among the three groups of patients (log-rank test, *p* = 0.15) (Figure 1).

## Discussion

To our knowledge this is the first report on the clinicopathologic features of breast cancer in pregnant and lactating Taiwanese women. We also evaluated predicting factors for delayed treatment in pregnant women with breast cancer. Our study design used an appropriate comparison group of non-PABC patients to evaluate PABC patients' demographic, clinical characteristics, tumor hormone-receptor status (ER, PR, and HER-2/neu), treatment delay, and 5-year survival rate.

The major finding from this study was that a younger age at diagnosis, larger tumor size and more advanced cancer stage were noticed in patients with PABC compared to non-PABC. Pregnancy was a strong predictor of having a greater risk to delay initial treatment in patients with PABC. As such, the time from self-reported onset of symptoms to diagnosis (median = 3 weeks) and from diagnosis to definitive treatment (median = 1 week) was generally delayed, taking a median of 4 weeks (range = 1∼59 weeks) out of concern for the safety of the fetus compared with the non-PABC controls (median = 1 week), despite the theoretical goal that breast cancer treatment should not be delayed due to pregnancy [13], [24]. Our findings supported that the delay in detecting and diagnosing PABC was due to marked hypertrophy of mammary gland tissues during pregnancy and lactation [25].

Prompt attention to the delay between subjective symptom reporting and the start of treatment is clinically indicated, as it impacts patient survival. Whether these delays were due to the patient's psychosocial factor or the physician's factor should be further investigated, but findings suggest that under 35 years old and nulliparous women both largely ignored the abnormal breast changes unless a lump was present; while early cancer stage (stage I) was also a predicting factor for delaying initiation of treatment after diagnosis. It was found in this study, that the risk of delaying initial treatment was greater for PABC patients whose breast cancers were classified as stage I. Previous studies noted that physicians were able to recognize women with more malignant cancers, thus the patients with longer delays suggested by doctors were those that had tumors that were in early stages and had less chance of spreading and metastasis than those with shorter delays ordered by doctors [26]. The PABC patients of the antepartum subgroup had a longer delay from diagnosis to initial treatment compared to that of the postpartum subgroup with shorter delays may have been due to pregnancy itself. Because these patients usually postponed treatment in consideration of fetal lung maturation and well-being [5], [9], [27]. However, our results also showed that there was no definitive association between antepartum PABC patients' presurgical neoadjuvant chemotherapy/mastectomy and fetal compromise or preterm labor [7], [11], [12], [21], [28]. Taken together, these results suggest that because cancer during pregnancy is a stressful situation, therefore, physicians and other health care professionals should encourage pregnant patients with PABC to begin treatment as soon as possible. Consistent with this finding, an expert meeting made the recommendation that all women who are pregnant or 35 years and older, who are considering to become pregnant should have a clinical breast examination as part of the prenatal routine visit for early diagnosis PABC [13].

In this study, we found that PABC shared many histological characteristics with non-PABC tumors except size, cancer stage, and progesterone receptor expression. Notably, we found no significant difference in terms of 5- year overall survival in PABC patients and non-PABC patients, which is similar to the previous large, population-based retrospective evaluation studies comparing PABC with non-PABC matched for stage and age [9], [10], [14], [15], [19]–[21], [29]. As previously studies showed, the risk factors affecting the survival of breast cancer patients were: women with a recent pregnancy [18], or a definitive treatment delay of 3-6 months [30]. Our data showed that there was a delay in diagnosis and initial treatment of the pregnant patients who were under 3 months (median = 8 weeks) of gestation, but resulted in no difference in the 5-year survival compared with the postpartum subgroup and non-PABC controls. However, pregnant or breastfeeding women have higher serum levels of estrogen and progesterone which may contribute to the high metastatic rate of breast tumor that is diagnosed shortly after pregnancy [6], [29]. Although hormone receptor's distribution of PABC has not been well studied [31], a previous study showed that PABC patients were more likely to be PR-negative and have a higher histological grade [32]. The present study also showed that there were less PR-positive tumors in PABC patients than non-PABC. While the proportion of ER-positive and both ER and PR-positive tumors were the same in PABC and non-PABC controls. This finding appears to differ from previous reports, primarily from non-Asian countries, that ER-negative tumor were more common in PABC women than non-PABC women [9], [12], [27], [29]. This difference in hormone receptor may come from sample population's difference in race or sample bias. Over-expression of the HER-2/neu oncogene, which occurs in 20 to 30% of invasive breast cancers, is associated with poorer prognosis [33]. HER-2/neu is synthesized in many embryonic tissues during pregnancy [34], and thus HER-2/neu over-expression was observed in 47.6% of the PABC patients, which is higher than that reported by Middleton (28%) [32], Garcia-Manero (31.6%) [12] and Halaska (33.3%) [17]. The rate of HER-2/neu oncogene over-expression in PABC patients was also higher than that among non-PABC group, which is similar to previously reported studies of 44% versus 28% of the control group [35].

This study had both advantages and limitations in comparison with previous reports of PABC. This is the first preliminary study to report PABC patients' treatment regimen and survival outcomes in Taiwan. As such, this study's strength is the collection of reproductive and cancer-treatment data for all patients, which were completely ascertained and provide information on a wide range of potential risk factors, clinical presentation, mode and timing of diagnosis and treatment, tumor characteristics as well as prognosis. We found that breast cancer patients in Taiwan tend to be younger than those from Western countries, which presents a different disease profile compared to previously published data. However, our study also had important limitations to consider. Firstly, the result of this study should be interpreted cautiously due to the retrospective nature and difference in follow-up time between PABC patients and non-PABC patients. Secondly, the total sample of 130 breast cancer patients (26 PABC and 104 non PABC) was small and patients were all from one teaching hospital in northern Taiwan which limits the generalizability of our findings. Thirdly, given the exploratory nature of this pilot study, potential correlations between the delay in treatment and the psychological impact of the PABC confirmation were not taken into account. We also incomplete information of non-PABC patients' lifestyle and environmental risk factors such as age at menarche, parity, age of first and subsequent pregnancies, breastfeeding, body mass index (BMI), weight gain and alcohol consumption that may possibly the adverse prognostic factors and an impact on the survival, to compare the differences between the PABC and non-PABC patients. Whether these risk factors affect survival or alter the impact on PABC and non-PABC, it should be investigated by further studies with a prospective study design. Nevertheless, compared with non-PABC controls, diagnostic delays and treatment delays have been noticed in the pregnant patients with abnormal breast symptoms and attention is needed to estimate the potential lifestyle and psychosocial risk factors which affect survival after a PABC diagnosis.

Based on the results of this study, we conclude that pregnancy alone does not result in a poor prognosis for PABC patients. However, the delays in diagnosis and initial treatment should be kept to a minimum. Considering the trend of delaying pregnancy into later reproductive years continues in Taiwan, we recommend that the physician or nurse practitioner work at OBGY clinics should be provided breast cancer screening combined the physical and ultrasound examinations to minimize the diagnosis delay. Once a pregnant woman is confirmed with breast cancer should be referred a comprehensive multidisciplinary and personalized approach to manage their treatment, which takes into account the health of both mother and fetus.
